# Genetics of Fusarium Wilt Resistance in Pigeonpea (*Cajanus cajan*) and Efficacy of Associated SSR Markers

**DOI:** 10.5423/PPJ.OA.09.2015.0182

**Published:** 2016-04-01

**Authors:** Deepu Singh, B. Sinha, V. P. Rai, M. N. Singh, D. K. Singh, R. Kumar, A. K. Singh

**Affiliations:** 1 Department of Genetics and Plant Breeding, Institute of Agricultural Sciences, Banaras Hindu University, Varanasi-221 005, Uttar Pradesh, India; 2 Agricultural Research Station, Tanchha, Bharuch, Navsari Agricultural University, Navsari- 396 450, Gujarat, India; 3 College of Agriculture and Research Station, Korea- 497 335, Chhattisgarh, India

**Keywords:** gene action, inheritance, validation, resistance breeding, SSR marker, wilt

## Abstract

Inheritance of resistance to Fusarium wilt (FW) disease caused by *Fusarium udum* was investigated in pigeonpea using four different long duration FW resistant genotypes *viz*., BDN-2004-1, BDN-2001-9, BWR-133 and IPA-234. Based on the F_2_ segregation pattern, FW resistance has been reported to be governed by one dominant gene in BDN-2004-1 and BDN-2001-9, two duplicate dominant genes in BWR-133 and two dominant complimentary genes in resistance source IPA-234. Further, the efficacy of six simple sequence repeat (SSR) markers namely, ASSR-1, ASSR-23, ASSR-148, ASSR-229, ASSR-363 and ASSR-366 reported to be associated with FW resistance were also tested and concluded that markers ASSR-1, ASSR-23, ASSR-148 will be used for screening of parental genotypes in pigeonpea FW resistance breeding programs. The information on genetics of FW resistance generated from this study would be used, to introgress FW resistance into susceptible but highly adopted cultivars through marker-assisted backcross breeding and in conventional breeding programs.

Pigeonpea (*Cajanus cajan* L. Millspaugh) is a major legume crop of the tropical and subtropical regions. It is a diploid species (2*n* = 2*x* = 22) comprising a genome of 833.1 Mbp arranged into 11 linkage groups (Varshney et al., 2012). India is the centre of origin and largest producer of pigeonpea in the world sharing approximately 70% of the production and covering 74% of the area (Bohra et al., 2012). It plays an important role in food security, balanced diet and subsistence agriculture because of its diverse usages in food, fodder, fuel, soil conservation, integrated farming systems and symbiotic nitrogen fixation (Reddy et al., 2005).

Fusarium wilt (FW), caused by fungal pathogen *Fusarium udum*, is one of the major disease widely prevalent in north and central parts of the India causing yield loss ranging from 30 to 100% (Reddy et al., 1990). The yield loss due to this disease also depends upon the stage at which the plant wilt and it can approach over 50% and even up to 100% when wilt occurs at the pre pod stage (Okiror, 2002). FW is a serious disease in South Asia and other parts of the world e.g., Kenya, Malawi. It is also reported from Bangladesh, Mauritius, Ghana, Tanzania, Uganda, Indonesia, Thailand and Trinidad (Nene, 1980).

The disease is soil and seed borne therefore, difficult to manage through fungicide alone. Continuous use of fungicides results in detrimental effect on environment and development of resistant strains of the pathogen. One of the best possible ways to reduce yield losses due to FW is to grow resistant pigeonpea varieties. Therefore, enhancement of resistance to FW in pigeonpea is a major challenge, which needs to be addressed on priority basis. A thorough knowledge of the inheritance of FW resistance in pigeonpea will be useful in initiating an effective breeding programme (Zhang et al., 2007). Several studies have been conducted to understand the genetic systems that control wilt disease in pigeonpea but, conclusive evidence is yet to arrive about genetics of FW resistance in long duration pigeonpea.

Molecular markers served several functions in pigeonpea including, genetic diversity analysis (Odeny et al., 2007, Singh et al., 2013) characterization of hybrid parents and purity assessment (Saxena et al., 2010), mapping for drought tolerance (Saxena et al., 2011), determinacy (Mir et al., 2012), sterility mosaic disease (Gnanesh et al., 2011) and association of SSR markers with FW resistance (Singh et al., 2013). The present study is an attempt to understand the genetics of resistance to FW in long duration pigeonpea and to validate the SSR markers associated with FW resistance in order to check their efficacy in pre-screening of diverse parental lines used in FW resistant breeding programs.

## Materials and Methods

### Plant material

Four long duration FW susceptible pigeonpea genotypes namely, BAHAR, MA-6, MAL-13 and MAL-18 and four FW resistant genotypes namely, BDN-2004-1, BDN-2001-9, BWR-133 and IPA-234 were selected for the present study (Table 1). All the genotypes were sown in crossing blocks at Agricultural Research Farm, Institute of Agricultural Sciences, Banaras Hindu University, Varanasi, India during rainy season 2011–12. The different cross combinations were made to obtain twelve F_1_ hybrids as presented in table 2. The twelve F_1_ along with parents were grown in crossing blocks during rainy season 2012–2013 and each F_1_ plant was allowed selfing in a mosquito-net protected field to avoid out crossing, simultaneously fresh F_1_s were also made to grow parents, F_1_s and their respective F_2_ populations in the same year i.e., rainy season 2013–2014.

### Fusarium wilt screening

One row plot of each of the parents, two rows of each F_1_s, and eight rows of each F_2_s were grown in compact family block design in wilt sick field plot at Agricultural Research Farm, Institute of Agricultural Sciences, Banaras Hindu University, Varanasi, India during rainy 2013–2014. As per availability of the seeds, the plot size of few segregating F_2_ populations was reduced. Each plot consisted of one row of 3 meter length with spacing of 75 × 25 cm between and within rows, respectively. Recommended agronomic practices were followed to raise a good crop. Chopped wilted pigeonpea plant stems (5–8 cm long) were uniformly buried into the soil across the field every year to artificially enhance and maintain the *F. udum* inoculum load (5 × 10^6^ spores/m^2^). To access the uniformity of disease incidence, one infector row of susceptible check ‘Bahar’ was planted after every 10 rows of test genotypes. The scoring of the susceptible (completely or partially wilted) and resistant (wilt free) plants of each F_2_ population of each crosses were done twice i.e., at the time of pod initiation as well as during pod maturity. Chi-square (χ^2^) test was applied to assess the goodness of fit to appropriate genetic ratio for the estimation of number of gene(s) governing FW resistance.

### DNA amplification

Young leaf tissues from 15-days old plantlets were collected from eight pigeonpea genotypes and stored at −20°C till DNA extraction. The genomic DNA was extracted using the Geneaid’s Genomic DNA Mini Kit (Biochem Life Sciences, New Delhi, India). In order to check the integrity of the genomic DNAs, 3–5 μl samples of the genomic DNAs along with the gel loading dye were individually loaded on to an ethidium bromide stained 0.8% agarose (Fischer Scientific, Pittsburgh, USA) gel. After 2 hr of electrophoresis in 1× TAE buffer, the gel was visualized under UV light. A thick band without any smear in the upper part of gel was indicative of high molecular weight and good quality genomic DNA. A total of six pigeonpea SSR markers, namely ASSR-1, ASSR-23, ASSR-148, ASSR-229, ASSR-363 and ASSR-366, were selected from the work of Singh et al. (2013) on association of pigeonpea specific SSR markers with FW resistance (Table 3). Polymerase chain reaction (PCR) reaction mixture (15 *μ*l) consisted of 20–25 ng of genomic DNA, 200 *μ*M dNTPs, 2 mM MgCl_2_, 1 unit *Taq* DNA polymerase (MBI Fermentas, Hanover, USA), 1× PCR buffer and 0.6 mM reverse and forward primers. DNA amplification was carried out in a Thermal Cycler (Mastercycler gradient, Eppendorf, Hamburg, Germany) with a PCR profile which included an initial denaturation step at 94°C for 3 min followed by 35 cycles with a denaturing step at 94°C for 30 s, a primer annealing step at optimum annealing temperature for 30 s and an extension step at 72°C for 1 min. After the last cycle, samples were kept at 72°C for 5 min for final extension. The amplification products were separated electrophoretically in 2.5% agarose gels containing 0.05 *μ*g/ml ethidium bromide and prepared in 1× TAE buffer. The amplification products were examined under UV light and photographed using a gel documentation system (Gel DocTM XR+, Biorad Laboratories, Hercules, USA). SSR banding profile from only that genotype × primer combination, which gave consistent amplification for all the genotype and without any blank lane/unclear bands, was included in this study. The amplified fragments were scored as ‘+’ for the presence of a band specific to Fusarium wilt susceptible check ‘BAHAR’ and ‘−’ indicates presence of a band at different position than in ‘BAHAR’.

## Results and Discussion

### Genetics of Fusarium wilt resistance

All the F_1_s resulting from twelve crosses, involving 4 R × 4 S pigeonpea genotypes were exhibited resistant reaction to FW, indicated dominance of resistance over susceptibility (Table 2). The F_2_ population of the crosses BAHAR × BDN-2004-1, MA-6 × BDN-2004-1, MAL-13 × BDN-2004-1 and MAL-18 × BSMR-846 (χ^2^ = 0.15–3.49; P = 0.062–0.700) segregated into 3R:1S genetic ratio indicating monogenic (one dominant) control of FW resistance in these crosses. These results substantiated that the resistant parent BDN-2004-1 possessed one major dominant for resistance. Similarly, three crosses *viz*., BAHAR × BDN-2001-9, MA-6 × BDN-2001-9 and MAL-13 × BDN-2001-9, involving the resistant parent BDN-2001-9 also exhibited monogenic control of resistance (Table 2). Dominant control of FW resistance was also reported by a number of workers (Changaya et al., 2012; Joshi, 1957; Pandey, 1996; Saxena et al., 2012). The dominant nature of inheritance will offer ease in incorporation of FW resistance from resistant to susceptible cultivars with any selection method (Pastor-Corrales et al., 1994). Recently, Changaya et al. (2012) reported involvement of single dominant gene in Malawian pigeonpea genotypes crossed to FW R-donors.

The F_2_ population of the crosses BAHAR × BWR-133, MA-6 × BWR-133 and MAL-13 × BWR-133 segregated with a good fit to 15R:1S. It confirmed that two duplicate dominant resistance genes governed the resistance in these three crosses (Table 2). Thus, it is evident that resistant parent BWR-133 possessed two duplicate dominant genes for FW resistance. The 15R:1S ratio suggested that two independent dominant genes with equal effects confer resistance to FW (Singh, 2005). The F_2_ population of the crosses BAHAR × IPA-234 (χ^2^ = 1.45; P = 0.0.229 and MAL-13 × IPA-234 (χ^2^ = 0.50; P = 0.480) segregated with a good fit to 9R:7S (Table 2). It confirmed that two complementary dominant resistance genes governed the resistance in these two crosses. Complementary gene actions for resistance to FW in pigeonpea have also been reported by Singh (2005) and Okiror (2002). It is concluded that in IPA-234 two pairs of dominant genes governed the resistance. Involvement of two or more genes as against monogenic control of FW resistance reported earlier (two independent dominant genes by Singh, 2005; two complementary genes by Okiror, 2002, minor polygenes by Sharma, 1986). Involvement of one or more recessive genes for control of FW resistance has also been reported by some researchers (Jain and Reddy, 1995; Odeny et al., 2009).

### Efficacy of SSR markers to distinguish parents involved in different crosses

**Fig. 1:**
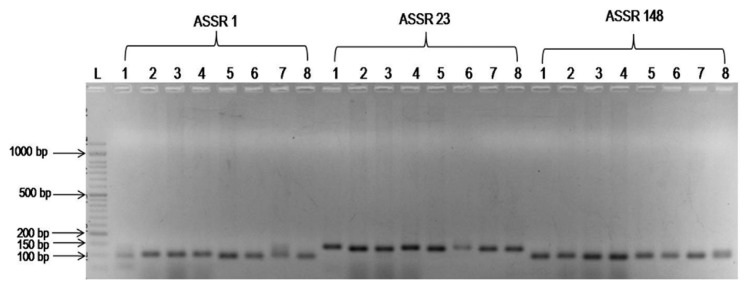
PCR banding pattern of the SSR markers ASSR 1, ASSR 23 and ASSR 148 associated with FW resistance. L = 50 bp DNA ladder; 1–8 (pigeonpea genotypes as listed in Table 1).

**Fig. 2:**
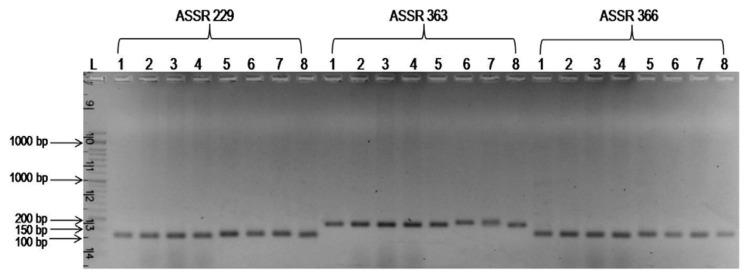
PCR banding pattern of the SSR markers ASSR 229, ASSR 363 and ASSR 366 associated with FW resistance. L = 50 bp DNA ladder; 1–8 (pigeonpea genotypes as listed in Table 1).

In order to determine the utility of molecular markers associated with the FW resistance, four FW resistant and four susceptible pigeonpea genotypes were screened with six SSR markers associated with FW resistance (Singh et al., 2013). SSR marker ASSR 1 amplified a fragment of 120 bp in ‘BAHAR’ and other three FW susceptible genotypes *viz*., MA-6, MAL-13 and MAL-18 (Table 4, Fig. 1). Whereas, an amplification product of 100 bp was found in three of the four FW resistant genotypes except, BWR-133. Similarly, SSR marker ASSR 148 amplified a 100 bp fragment in all FW susceptible genotypes except, MAL-18 and 110 bp amplification product in all FW resistant genotypes except, BWR-133. An amplification product of 150 bp was produced by marker ASSR 229 in FW susceptible genotypes but ‘BAHAR’ unable to amplify it and produced a fragment of 135 bp (Table 4, Fig. 2). It was interesting to note that marker ASSR 366 uniformly produced a band of 120 bp in all the FW susceptible and resistant pigeonpea genotypes except, two resistant genotypes (BDN-2001-9 and IPA-234).

Singh et al. (2013) studied the association of SSR markers with FW resistance by using a diverse set of 36 pigeonpea genotypes. Kruskal-Wallis one-way analysis of variance (K-W ANOVA) detected the significant association of six SSR markers *viz*., ASSR-1, ASSR-23, ASSR-148, ASSR-229, ASSR-363 and ASSR-366 with Fusarium wilt resistance. The same six markers also showed significant association in simple regression analysis owing to higher R^2^ values and significant deviation of b value from zero. Among the six markers, ASSR-363 explained a maximum of 56.4% (b value = 1.86; *P* < 0.01) of phenotypic variation due to FW resistance. The phenotypic variation explained by these six markers ranged from 23.7 to 56.4%. Earlier, Mace et al. (2006) identified association of eight SSR markers with rust and late leaf spot (LLS) using K-W ANOVA in groundnut.

The result of the amplification product on the basis of presence/absence of a band specific to FW susceptible check ‘BAHAR’ have been shown in Table 4. Marker ASSR 1 was able to identify 9 out of 12 cross combinations made in the present study (Table 5). Similarly, parents involved in 8 cross combination will be distinguished by three of the SSR markers used in the present study i.e., ASSR 23, ASSR 148 and ASSR363 (Table 5). While, ASSR 366 was only identify five crosses out of a total of 12 cross combinations made in the present investigation. On the basis of differential amplification of six SSR markers used in the present study, markers ASSR 1, ASSR 23 and ASSR 148 were found to be most efficient in parental polymorphism screening of the crosses made between diverse FW susceptible and resistant pigeonpea genotypes.

In conclusion, the present study has used four different FW resistant pigeonpea genotypes (BDN-2004-1, BDN-2001-9, BWR-133 and IPA-234) with diverse backgrounds. FW resistance of these genotypes has been reported to be governed by one dominant gene (BDN-2004-1 and BDN-2001-9), two duplicate dominant genes (BWR-133) and two dominant complimentary genes (IPA-234). Utility of six SSR markers namely, ASSR-1, ASSR-23, ASSR-148, ASSR-229, ASSR-363 and ASSR-366 reported to be associated with FW resistance, were also tested and found that ASSR-1, ASSR-23, ASSR-148 will be used for screening of parental genotypes in pigeonpea FW resistance breeding programs. The information on genetics of FW resistance generated from this study would be used, to introgress FW resistance into susceptible but highly adopted cultivars through marker-assisted backcross breeding and in conventional breeding programs.

## Figures and Tables

**Table 1:** List of the resistant and susceptible pigeonpea genotypes used for crossing program

S. N.	Genotype	Pedigree	Source	Characteristic features
1.	Bahar	Selection from Motihari district, Bihar, India	RAU, TCA, Dholi, Bihar, India	Medium height, compact, yellow flower, purple pod containing medium brown seed
2.	MA-6	MA-2 × Bahar	BHU, Varanasi, Uttar Pradesh, India	Semi-spreading, yellow flower, purple pod, highly resistant to Sterility Mosaic Virus
3.	MAL-13	(MA-2 × MA-166) × Bahar	BHU, Varanasi, Uttar Pradesh, India	Spreading, light yellow flower, pod long, green, constricted with purplish black streaks containing large brown seed (13 g/100 seeds) and moderately resistant to Sterility Mosaic Virus
4.	MAL-18	MA-2 × *Cajanus cajanifolius*	BHU, Varanasi, Uttar Pradesh, India	Spreading, yellow flower, purple pod, highly resistant to Sterility Mosaic Virus
5.	BDN-2004-1	Mutant of BSMR-853	ARS, Badnapur, Jalana, Maharastra, India	Semi spreading, medium dwarf having purplish stem and flower, pod green with purplish streaks, seed dull white, 8.5 g/100 seed and resistant to wilt
6.	BDN-2001-9	BDN-2 × BWR-370	ARS, Badnapur, Jalana, Maharastra, India	Spreading, yellow flower, pod green with streaks, seed brown with white hilum,11.2 g/100 seeds and resistant to wilt
7.	BWR-133	–	ARS, Badnapur, Jalana, Maharastra, India	Semi spreading, yellow flower, pod green with streaks white seed, 9.6/100 seeds and resistant to wilt
8.	IPA-234	–	IIPR, Kanpur, Uttar Pradesh, India	Compact, yellow flower, pod green with streaks and resistant to wilt

**Table 2:** FW reaction of nine F_2_ populations of pigeonpea derived from crosses between three resistant and three susceptible genotypes

Cross	F_1_	No. of F_2_ plants		Total	Expected ratio (R:S)	χ^2^ value	P-value

		R	S				
BAHAR × BDN-2004-1	R	150	39	189	3:1	1.81	0.179
MA-6 × BDN-2004-1	R	178	43	221	3:1	3.49	0.062
MAL-13 × BDN-2004-1	R	104	37	141	3:1	0.15	0.700
MAL-18 × BDN-2004-1	R	107	25	132	3:1	2.59	0.107
BAHAR × BDN-2001-9	R	108	25	133	3:1	2.58	0.108
MA-6 × BDN-2001-9	R	76	18	94	3:1	1.72	0.190
MAL-13 × BDN-2001-9	R	116	29	145	3:1	1.81	0.179
BAHAR × BWR-133	R	181	17	198	15:1	2.22	0.136
MA-6 × BWR-133	R	78	8	86	15:1	1.91	0.167
MAL-13 × BWR-133	R	163	9	172	15:1	0.39	0.532
BAHAR × IPA-234	R	44	26	70	9:7	1.45	0.229
MAL-13 × IPA-234	R	69	61	130	9:7	0.50	0.480

**Table 3:** Detail of the SSR markers used in the present study

Marker	Forward sequence	Reverse sequence	(SSR motif)_n_	Tm (°C)	Observed size range (bp)	No. of alleles
ASSR-1	GTCCGTTGAAAAACAAAGAG	CGTTTTAGGTTTCTTCTCTGC	(GA)_10_	55	100–120	2
ASSR-23	CTTTCCCTTCTCTCTCAACAC	AAGCAGAAGCAGAAGCAGAG	(CCTTCT)_5_	55	120–160	2
ASSR-148	AACCGATGCTTTCTTCTACTAC	ACTCAACGGTGCTACTCATC	(CAA)7	55	140–160	2
ASSR-229	ATAGTGGGACAGTAGAAAATCC	CAACTCATCTCTTGGTTCTCC	(TAAGGG)_5_	55	150–160	3
ASSR-363	GGGAGAAGTATAAGGAGAAATG	TCACCCTTTGATAATGTTCC	(GCATCA)_5_	55	190–210	2
ASSR-366	CTCTGCAACTCGCTCATTTC	ACGTGATGGAGAAGATCCAAC	(CGT)_8_	55	140–180	2

**Table 4:** Size of amplification product in eight pigeonpea genotypes using SSR markers associated with Fusarium wilt (FW) resistance

Genotype	FW reaction	Approximate size of amplification product (bp)					

		ASSR 1	ASSR 23	ASSR 148	ASSR 229	ASSR 363	ASSR 366
BAHAR	S	120 (+)	150 (+)	110 (+)	150 (+)	200 (+)	135 (+)
MA-6	S	120 (+)	135 (−)	110 (+)	135 (−)	200 (+)	135 (+)
MAL-13	S	120 (+)	135 (−)	110 (+)	135 (−)	170 (−)	135 (+)
MAL-18	S	120 (+)	150 (+)	100 (−)	135 (−)	200 (+)	135 (+)
BDN-2004-1	R	100 (−)	135 (−)	100 (−)	150 (+)	170 (−)	135 (+)
BDN-2001-9	R	100 (−)	150 (+)	100 (−)	135 (−)	170 (−)	120 (−)
BWR-133	R	120 (+)	135 (−)	110 (+)	150 (+)	170 (−)	135 (+)
IPA-234	R	100 (−)	135 (−)	100 (−)	150 (+)	200 (+)	120 (−)

Sign within parentheses indicates the presence (+)/absence (−) of a SSR band. ‘+’ indicates presence of a band specific to Fusarium wilt susceptible check, BAHAR and ‘−’ indicates presence of a band at different position than in BAHAR.

**Table 5:** Summary of parental polymorphism using SSR markers among different crosses studied in present investigation

Cross	SSR markers associated with FW resistance					

	ASSR 1	ASSR 23	ASSR 148	ASSR 229	ASSR 363	ASSR 366
BAHAR × BDN-2004-1	✓	✓	✓	X	✓	X
MA-6 × BDN-2004-1	✓	X	✓	✓	✓	X
MAL-13 × BDN-2004-1	✓	X	✓	✓	X	X
MAL-18 × BDN-2004-1	✓	✓	X	✓	✓	X
BAHAR × BDN-2001-9	✓	X	✓	✓	✓	✓
MA-6 × BDN-2001-9	✓	✓	✓	X	✓	✓
MAL-13 × BDN-2001-9	✓	✓	✓	X	X	✓
BAHAR × BWR-133	X	✓	X	X	✓	X
MA-6 × BWR-133	X	X	X	✓	✓	X
MAL-13 × BWR-133	X	X	X	✓	✓	X
BAHAR × IPA-234	✓	✓	✓	X	X	✓
MAL-13 × IPA-234	✓	X	✓	✓	✓	✓

Total	9/12	6/12	8/12	7/12	9/12	5/12
